# Evaluation of resources for contact lens practice in private contact lens clinics of Muscat, Oman

**DOI:** 10.4103/0974-620X.48417

**Published:** 2009

**Authors:** Rajiv Khandekar, Mohammed Al Fahdi

**Affiliations:** Eye and Ear Health Care, Department of Control of Non Communicable Disease, DGHA, Ministry of Health, Oman

**Keywords:** Contact lens, corneal blindness, health facility assessment, prevention of blindness, refractive error

## Abstract

**Background:**

The integration of the contact lens (CL) practice with the VISION 2020 initiative is important. We assessed the facilities at the private CL clinics of Muscat. Accordingly, we suggested the appropriate eye care for CL wearers in Oman.

**Study Design:**

This was a descriptive study.

**Materials and Methods:**

This study was conducted between May and July 2006. A team of optometrists and health inspectors visited clinics and collected information about the infrastructure, available human resources, and materials used in the CL practice. We used a pre-tested close-ended questionnaire to collect responses of the practitioners and observations of the field staff.

**Statistical Method:**

Univariate parametric type of analysis.

**Results:**

The team visited 67 CL clinics and interviewed 75 CL practitioners. Proper hand washing facility was available at 61 clinics. Thirty-nine practitioners had >10 years of experience in dispensing contact lenses. Only 13 clinics had a bio-microscope. None of the clinics had legal documents signed by both providers and end users of the contact lens.

**Conclusions:**

Contact lens has received less attention in areas outside the developed world. The CL practice in the private sectors of Oman needs to be strengthened. Minimum standards, standard operating procedures for CL practice, and its periodic supervision would be useful.

## Background

Optometrists are important eye care providers. They dispense visual aids as well as act as a connecting link between eye patients with minor eye problems and ophthalmologists. A national VISION 2020 initiative should therefore include their activities within the purview of the program management.[1]

The Sultanate of Oman is a country in Middle Eastern peninsula with population of 2.5 million. Seventy- five percent of its population is of <40 years of age.[2] The prevalence of myopia ranges from 4.1% in 12-13 year old children to 8.8% in 16-17 year old children.[3] The compliance of use of spectacles among these children was 73% in one of the rural region of Oman.[4] Contact lens (CL) users are on the rise in Oman. The trend in urban population of Oman is changing as they prefer contact lenses instead of spectacles.

Contact lens services are at present with the private sector and it is yet to be standardized. Administrators of the Ministry of Health (MOH) are aware of this challenge and aim to ensure better governance of the CL practice. The department of ‘Private Health Establishment’ issues a license to qualified optometrists to practice in optical shops. Qualified senior ophthalmologists assess their caliber prior to their registration. However, an optician who has license to practice in an optical shop does not have to undergo any special formalities and tests to start a CL practice.

Ophthalmologists, especially the cornea specialists have noted complications like chronic papillary hypertrophy, bacterial and Acanthamoeba keratitis that could be linked to the CL practice (Bialasiewicz AA. 1^st^Contact lens Conference, Muscat, Oman, 2004). Although CL related ocular complications could be one of the underlying causes of corneal opacities - a priority eye problem in ‘VISION 2020 - OMAN,’ its contribution is not known.[5] Making CL providers and end users aware of CL related complications will reduce possibility of such catastrophes. The 1^st^National Conference of Contact Lens was organized by the ‘Eye Health Care Program’ in Muscat in 2004 and aimed to increase awareness of optometrists about current trends of CL practice and MOH’s expectations of their role as eye care providers.

The MOH planned to conduct a situation analysis of CL practice in Oman. Since majority of CL clinics were located in Muscat city, it was decided to begin the review in Muscat region. In 2006, selected staff of the ‘Eye Health Care’ and the ‘Department of Private Establishment,’ visited private optical shops and evaluated the facilities for CL practice. The authors present the profile of the resources in the CL clinics of Muscat region.

## Materials and Methods

This descriptive study was conducted between May and July 2006, and approved by the National Eye Health Care committee and the MOH. National supervisors of ‘Eye Health Care’ and the ‘Department of private establishment,’ which maintain the registry of optical shops, were our field investigators. The registry was reviewed and CL clinics were identified. A letter outlining the purpose of the visit and requesting the optometrists’ cooperation to generate reliable information was issued to all optical shops. A standard form was prepared, tested, and used for this purpose [Table 1]. The same team visited all the CL clinics. They spent between 45 minutes to one hour in each CL clinic to undertake this assessment. They explained the purpose of the visit and took the consent of the practitioner to participate in the study. Part (A) was to get information on the practitioner and facilities available in the clinic. Part (B) was related to the observations of the team members. The feedback was provided to the head of the CL clinic. The data of the form was entered using EPI6 software. Univariate analysis was carried out by parametric method using Statistical Package for Social Studies (SPSS-9). The numbers and percentage proportions were calculated.

**Table 1:** Contact lens clinic resource assessment form

*Title*	*Response*
Name of the optical shop	
License #:	
Address:	
Tel and Fax.	
A:	Details of contact lens practitioner: (Response of the participants)
	Name
	License number:
	Name of country where they trained for optometry
	What degree was awarded
	Duration of training
B:	Infrastructure in the contact lens clinic. (By observation)
	Number of rooms:
	For eye examination
	Counseling and waiting area
	Availability of hand washing facilities for practitioner
C:	Availability and condition of the equipment
	Phoropter:
	Auto-keratorefractometer:
	Manual keratometer:
	Retinoscope and trail lens set:
	Lensometer:
	Slit lamp: Bio-microscope
	Others (Specify):
D:	Type of contact lenses dispensed
	Soft contact lenses
	Gas Permeable hard lenses
	Silicon lenses:
	Toric:
	ROSE-K
	Cosmetic
E:	Types of contact lens solutions (write all possible types available)
	Name
	Date of expiry
F:	Health education materials:
	Booklet
	Videos:
	Others
G:	Impression of cleanliness and hygiene:
H:	Record Keeping
	Lens specifi cations:
	Patient’s details
I:	Legal document:

## Results

**Profile of the study sample:** The team visited 67 CL clinics. Seventy-five opticians were working in these clinics and 65 participated in the study. Two practitioners were absent during the study period. However, information on the clinic could be collected partially from other staff in the clinic. Seventy-three opticians were registered with the health authorities. Two practitioners received the approval from the MOH recently but were awaiting registration. In fifty-eight clinics, only one optician provided services. In nine clinics, two opticians were present.

**Human resource for contact lens practice:** Of the 75 CL practitioners, 57 (76%) had a Diploma in Optometry, 17 (22.7%) graduated in optometry, while one was an ophthalmologist. The duration of CL practice is given in Table 2. More than half of the CL practitioners had more than ten years of experience in CL practice. The practitioners were trained in different institutions in six countries. Forty-six opticians were trained in India, fifteen in the Philippines and five each in Sudan and Jordan. Two opticians had qualification in Pakistan while one Omani optometrist was trained in UK.

**Table 2:** Experience of contact lens practitioners in Muscat, Oman

*Duration in years*	*Number of persons*	*Percentage*
<5	4	5.3
5 to 9	30	40.0
10 to 14	16	21.3
15 and more	23	30.7
Missing (absent during visit)	2	2.7
Total	75	

**Facilities in contact lens clinics:** A team member noted the facilities available in the clinics [Table 3]. A bio-microscope was available in one fifth of the clinics.

**Table 3:** Facilities in clinics

*Items*	*Clinics with facilities*	
	*Number*	*Percentage proportion*
Availability of water for washing hands	61	91.0
Phoroptor	38	56.7
Autokeratorefractometer	14	20.9
Autorefractometer	53	79.1
Manual keratometer	21	31.3
Direct retinoscopes	41	61.2
Trial of contact lens	63	94.0
Lensometer	67	100.0
Slit lamp	13	19.4
Ophthalmoscope	6	9.0
Hand held Magnifi er	7	10.4
Ophthalmic loupe	4	6.0

**Types of contact lenses and solutions dispensed:** All practitioners dispensed soft and cosmetic contact lenses. Toric lenses were available at 41 clinics. Hard, silicon, and ‘Rose K’ lenses were dispensed at fifteen clinics. A wide variety of CL solutions were used in these clinics [Table 4].

**Table 4:** Contact lens solutions used

*Contact solution*	*Clinics with brand of lens solution mainly dispensed*	
	*Number*	*Percentage*
Accusoft	1	1.5
Aryan	2	3.0
Bausch and Lomb	4	6.0
Biofresh	4	6.0
Bio-medics all in one	2	3.0
Boston	8	11.9
Carrera	1	1.5
Complete	17	25.4
Fashion care	4	6.0
Fresh look	13	19.4
Hypa oppia	5	7.5
I care	4	6.0
Ons merk1	2	3.0
Optifree	28	41.8
Perfect	4	6.0
Renu multiplus	14	20.9
Solo care	39	58.2
Total	6	9.0
Xpressions	17	25.4

**Health education material:** To improve the understanding about CL and reduce complications due to the misuse of the contact lens, counseling of the CL wearers is very important. For this purpose, various materials were used. Pamphlets/booklets on CLs were found in 58 clinics, while posters and magazines were displayed in 47 and 12 clinics, respectively. None of the clinics had video presentations on the safe use of CLs.

**Legal document and case records:** We did not find legal documents stating that the provider explained the risks, proper use, and care of CL, their responsibilities and had signatures of both the parties in any of the CL clinics. In 56 (83.6%) clinics, records of the CLs dispensed were maintained. In only 47 (70.1%) clinics, detailed information on clients, their refractive status, and behavior of CL during the trial were noted.

## Discussion

Cases of CL induced keratitis are on the rise in Oman. The profile of such patients at tertiary eye hospital suggested that early intervention and standard care could prevent severe visual disabilities.[6] But, as usual prevention is better than the cure. To prevent keratitis due to contact lens, they should be dispensed by qualified persons at dispensing units with standard facilities. Therefore, this study was vital.

The response rate of more than 95% of CL practitioners in the Muscat region of Oman was far better than the 21% reported in Hong Kong where ‘postal questionnaire method’ was used to collect the data.[7] The field staff in our study represented the MOH, which is the licensing authority in Oman. We attribute the high response rate in our study to prior communication with the clinics.

All clinics were dispensing soft and cosmetic contact lens. A high popularity of soft CL was also noted in other studies. 88% of the dispensed lenses in Hong Kong, and 93% of lenses in Australia were of soft variety.[7,8] Facilities and resources that are essential for a standard CL practice were not met in the study area. The MOH has proposed the minimum requirements for a standard CL clinic in Oman. (Al Raisi A. 3^rd^ Contact Lens Conference, Muscat, Oman, May 2007). We believe that confirmation of these standards by experts from cornea and CL subspecialties at the tertiary level hospitals in Muscat, Oman establishment of policies for CL practice in Oman would be beneficial. Periodic supervision could strengthen the eye care of CL wearers.

Since their introduction, the most significant complication of wearing soft CLs has been the development of vision-threatening microbial keratitis.[9] Complications due to the misuse of CLs can have disastrous consequences on CL wearers.[10] A CL practitioner should be able to identify early signs of corneal complications of CL. For this, a bio-microscope is an essential. Proper counseling and prompt reference of such cases to an ophthalmologist at an early stage can prevent sight-threatening complications. We recommend adverse event notification should be established for serious corneal complications of CLs within 24 hours. There should be a follow-up of such notified cases and the attending clinician should prepare a report based on the action taken.

Irregular and high astigmatism are mainly corrected by CLs. Information on these issues will enable to determine proportion of CL practice for therapeutic purpose and for cosmetic purpose. Keratoconus is very common in the children in Oman. There are 256 cases registered in a tertiary eye unit of Oman with keratoconus as principal diagnosis (Personal communication, Dept of Ophthalmology, Al Nahdha Hospital). These children will either need keratoplasty or toric CLs.

In establishing the liability of CL practitioner for negligence, the courts have applied the same standard of care that is imposed upon other health care professionals, holding that the optometrists must employ a certain degree of skill, care, and learning that is ordinarily exhibited by members of the profession who are in good standing.[11,12] Legal liability pertaining to complications of CLs is a serious issue and we found that the practitioners in Oman were not fully aware about it. The lens providers and customers do not sign legal contract. A standard legal document is routinely used and is strongly recommended by the American Optometry Association (AOA).[13] Contact lens practitioners should observe legal guidelines to minimize the risk of injury to their patients and to reduce the opportunity for litigation.[14]

Disposable and planned replacement lenses have become increasingly popular options for CL wearers. Cosmetic lenses could be sold as cosmetic devices in boutiques. Hence, Farkas has strongly suggested that optometrists devise management strategies to avoid problems that are caused by the use of these lenses.[15] However, quality control of such lenses and resulting eye complications will be difficult to supervise. Declaring CL as medical device which could be prescribed only by qualified persons is recommended in Oman to address this problem.

## Conclusions

This was an organized approach to integrate the contact lens (CL) practice within the VISION 2020 program in Oman. The present exercise will improve the standard of optical outlets that are offering CLs to the customers. The outcome of this study will also be of use to groups of international CL educators. Norms for CL practices with standard infrastructure requirements for CL clinics could be laid down in Oman and other countries with a similar situation of eye care. A sense of responsibility as a team member of eye health care should be embedded among CL practitioners. Information on the magnitude and grades of CL induced keratitis, to associate the available facilities to the blinding complications of CLs is required. Unfortunately, we do not have such information in Oman. A registry could be initiated and periodically reviewed as area of further research. These steps will be useful in improving quality of life of CL wearers.

## References

[1] Kocur I, Resnikoff S (2005). New challenges for VISION 2020. Ophthalmic Epidemiol.

[2] (2005). Director General of Health Affairs, MOH, Oman. Health Status Indicators. In: Annual Health Report year 2005, Al Zahra Printers, Muscat.

[3] Khandekar RB, Abdu-Helmi S (2004). Magnitude and determinants of refractive error in Omani school children. Saudi Med J.

[4] Khandekar R, Mohammed AJ, Al Raisi A (2002). Compliance of Spectacle wear and its determinants among schoolchildren of Dhakhiliya region of Oman: A descriptive study. SQU J Sci Res Med Sci.

[5] (2000). Ministry of Health, Sultanate of Oman. Eye Health Care Manual.

[6] Shah R, Shah M, Khandekar R, Al-Raisi A (2008). Contact lens induced corneal ulcer management in a tertiary eye unit in Oman: A descriptive study. SQU Med J.

[7] Cheung SW, Cho P, Edwards MH (2002). Contact lens practice in Hong Kong in the new millennium. Clin Exp Optom.

[8] Woods CA, Morgan PB (2002). Contact lens prescribing in the Australian states and territories 2001. Clin Exp Optom.

[9] Fleiszig SM, Evans DJ (2003). Contact lens infections: Can they ever be eradicated?. Eye Contact Lens.

[10] Mah-Sadorra JH, Yavuz SG, Najjar DM, Laibson PR, Rapuano CJ, Cohen EJ (2005). Trends in contact lens-related corneal ulcers. Cornea.

[11] Classe JG (1986). A review of professional liability cases affecting the practice of optometry. J Am Optom Assoc.

[12] Classe JG (1994). Avoiding liability in contact lens practice. Optom Clin.

[13] American Optometric Association. New Patient Information in. ‘Doctor eye care center’. http://www.aoa.org/x2363.xml.

[14] Miller PJ (1986). Liability issues in contact lens practice. J Am Optom Assoc.

[15] Farkas P (1994). Integrating disposable or planned replacement lenses into contact lens practice. Optom Clin.

